# “It’s not all nice and fun”: Narrating contested illness on YouTube and Instagram

**DOI:** 10.1177/13634593211017187

**Published:** 2021-05-25

**Authors:** Irene Groenevelt

**Affiliations:** Tilburg University, The Netherlands

**Keywords:** balanced positivity, contested illness, moral legitimacy, online ethnography, self-care practices, social media

## Abstract

This empirical study draws on insights from narrative theory to tease out how women with a contested illness narrate their experiences on social media. Based on 13 months of online observations between 2017 and 2019, I analyse how a sample of eight highly active Dutch female social media users share their illness on YouTube and Instagram. In addition, I interviewed five of them. Through their online performances, the women in this study illustrate their investment in self-care practices, whilst simultaneously laying bare the limits of these practices in ensuring permanent well-being. Central to transmitting their experiences is the performance of *balanced positivity*; meaning that illness is dealt with in a predominantly ‘positive’ way, as well as through the occasional display of (moments of) hardship. I identify three main aspects of this performance of balanced positivity, namely: (1) appearances, (2) mindset, and (3) presence. The practice of balanced positivity is congruent with the concept of legitimacy narratives, because it allows women with a contested illness to show their efforts to cope with their condition as well as the myriad challenges that remain despite these efforts.

## Introduction

In recent decades, the Internet has developed into a primary source of health and medical information (Barker, 2008). Scholars from various disciplines have sought to capture the dynamic ways in which users engage with digital technologies (Poletti and Rak, 2014; Van Dijck, 2009). Central to this newly emerging field of digital health is the idea that the development of the Internet has affected the subjective experience of illness (Keim-Malpass et al., 2014). Following Conrad et al. (2016), this is particularly true since the transition from Web 1.0 where users could browse through already existing content, to Web 2.0 where “the emphasis is on *user-generated* content and visibility” (p. 25, emphasis in original). This transition has enabled (chronically) ill individuals to share experiences and information, interact with others, and engage in various forms of advocacy in their preferred online environment(s). Accordingly, illness experiences have shifted from a private to an increasingly public affair (Conrad et al., 2016).

Several qualitative studies have shown how sufferers of contested illness make use of social media to strive for greater visibility and legitimacy, and do so by sharing personal experiences with others (Berard and Smith, 2019; Dumit, 2006; Phillips and Rees, 2017). These studies illustrate how social media participation can enhance knowledge exchange, stimulate peer support, and can offer a means for collective action. However, sharing contested illness online is also a risky endeavor. In their study on communication about invisible illness on social media, Sannon et al. (2019) claim that “stigma complicates identity negotiation and poses additional risk for disclosures in social media because people with stigmatized identities fear being misunderstood, stereotyped, blamed, and rejected” (p. 3). Users mitigate these risks by adopting impression management strategies, for example by being reserved about (directly) discussing their experiences. Sannon et al. (2019) further argue that the norm to post positive content that is prevalent on social media makes sufferers hesitant to open up about negative health-related experiences.

Building on and adding to this scholarship, the present study analyzes how Dutch women share their experiences of living with a contested illness on YouTube and Instagram. Following Marwick and Boyd (2010: 115), I understand identity presentation on social media to be shaped by an “imagined audience” that is based on technological affordances and the social context in which online practices are situated. The question that guides the analysis is: *How do social media users with a contested illness narrate their experiences on YouTube and Instagram?* The present study argues that *balanced positivity* is central to the online narratives of the women in this study. That is, they make use of YouTube and Instagram to illustrate their investment in self-care practices, while using these same media to draw attention to the limits of these self-care practices in ensuring wellbeing. These online narrations can be interpreted as *legitimacy narratives* through which social media users seek to affirm their moral legitimacy (Japp and Japp, 2005), and are interlinked with the present-day requirement of self-care in health.

The focus of this study is on experiences of living with chronic pain syndrome (CPS), fibromyalgia syndrome (FMS) and chronic Lyme disease (CLD). While CPS and FMS, in the Netherlands, are generally accepted diagnoses in mainstream medical practice, CLD (not to be confused with late-stage Lyme disease and post-treatment Lyme disease syndrome) is not (Marques, 2008; see also Willemsen, 2018). Most people who self-identify as CLD patients have received negative outcomes on regular diagnostic tests, suggesting to conventional medical practitioners that they have never been infected with Lyme disease (Lantos, 2015). Often they are diagnosed on the basis of non-standardized tests and treated in private clinics outside the Netherlands – including Belgium and Germany. Based on observations, it seems that – contrary to social media users with CPS and FMS – those with CLD are more inclined to use online platforms for crowd funding to finance (stem cell) therapy, and to use YouTube and Instagram to document their ongoing treatment trajectory. Symptomology can be similar to CPS and FMS, as is indicated by the fact that all respondents in this study with CLD received a CPS and/or FMS diagnosis prior to their current diagnosis.

## Narrations of contested illness

Typical narratives are characterized by “a discernable plot that has a beginning, middle and an end” (McMahon et al., 2012: 1359). In *The wounded storyteller*, Frank (1995) describes how the incorporation of a linear temporal sequence is a central element in two types of narratives, which he calls *restitution* and *quest*. Whereas restitution narratives express the drive to restore former health by getting proper care, quest narratives are all about the belief that valuable insights can be gained from the experience of being ill. Both types of narratives have a linear temporal structure: they start with the naming of the condition from which the protagonist is suffering, and work toward the restitution of health or the reconfiguration of identity. Because these narrative types are dominant in contemporary Western society, Wasson (2018) provocatively speaks of a “hierarchy of narratives” in which the incorporation of certain types of temporal structure has come to be understood as “indispensable to a bearable human life” (p. 107). Yet compared to illnesses that have a firmly established biomedical status, contested illnesses do not easily allow for the telling of such preferred narratives because, in most cases, there is no clearly demarcated beginning or ending of the suffering. This is why Nettleton (2006) argues that stories by people who lack a biomedical diagnosis can best be described as *chaos narratives*: Frank’s third and final typology. These stories are “difficult to ‘hear’” because they lack a linear structure and plot, and might even lead to the denial of the existence of the illness by others (Nettleton, 2006: 1173).

In various studies, the difficulties experienced in narrating contested illness have been connected to the contemporary imperative to health, conceptualized as the neoliberal understanding that health and wellbeing are individual accomplishments that can, and should, be safeguarded by appropriating and maintaining a healthy lifestyle (see Lupton, 1995). Often these studies refer to Crawford’s (1980) concept of *healthism*, which he describes as the tendency to situate “the problem of health and disease at the level of the individual” and to formulate solutions to ill health correspondingly. In their study on healthism in Denmark, Kristensen et al. (2016) write how healthism presses individuals to take “responsibility for their bodies by engaging in strict self-care regiments” (p. 488). They critically argue that healthism can generate moral judgments about those who are unwilling to abide to its imperatives. In the case of contested illness, moreover, being *unable* is readily mistaken for being *unwilling* to ascertain good health. In recent qualitative studies about contested illness and somatically unexplained symptoms, this sentiment is epitomized by the prevalence of accusations of laziness (Paxman, 2019), slackness (Østebye et al., 2018), and mental instability (Sowińska, 2018) by health-care providers and others. To counter such accusations, “questions and judgments regarding illness origin and the validity of complaints are often in the narrative forefront” (Swoboda, 2006: 235).

Feminist studies have further shown how psychological attributions are highly gendered, and how women are more readily denied the reality of their suffering than men are (Werner et al., 2004; Werner and Malterud, 2003). Based on qualitative interviews, Werner and Malterud (2003) illustrate how women with unexplained chronic pain have to work hard in order to be “believed, understood, and taken seriously when consulting a doctor” (p. 1409). They take care to express themselves in a proper way; one that involves not appearing “too strong or too weak, too healthy or too sick, too smart or too disarranged” (p. 1409). More generally, women with a contested illness anticipate challenges in bringing their stories across to others, and take care to convey their diligent attempts at overcoming chronic suffering (Paxman, 2019). Indeed, in her study on narratives of chronic fatigue syndrome, Bülow (2008a) argues that the very experience of being called into question is constitutive of the identity formation of women with a contested illness.

Along similar lines, Japp and Japp (2005) describe how the prevalence and persistence of medical and moral ambiguities surrounding contested illness seems to call for a certain type of narrative, which they label *legitimacy narratives*. Different from other illness stories, legitimacy narratives do not start with the naming of an illness, but rather with descriptions of the intensity and persistence of symptoms that testify to the realness of the suffering. However, such descriptions expose narrators to accusations of exaggerating, which reinforce disbelief, thereby presenting them with a “double bind” (p. 110). This study specifically focuses on the notion of moral legitimacy. Because contested illness does not generally result in “clear outward signs”, others often challenge its veracity, as well as the credibility of the sufferer (Sim and Madden, 2008: 62). Legitimacy narratives thus defend the moral character of the author by debunking the idea that physical symptoms are the result of psychological failing (Malterud, 2000), exaggerating or malingering (Japp and Japp, 2005). Extending the concept of legitimacy narratives to social media, the current study teases out how sharing contested illness takes place on YouTube and Instagram.

## Methods

The study consists of online observations and semi-structured interviews. After receiving approval of the Research Ethics Committee of Tilburg School of Humanities, I spent a total of 13 months (September 2017 to January 2018 and June 2018 to February 2019) conducting observations. Based on online searches and snowballing strategies I selected a purposive sample of 16 Dutch YouTubers who had uploaded one or multiple video(s) about their experiences of living with contested illness. During the first few interviews, I became aware of the importance of Instagram in many of the YouTubers’ digital lives. This finding informed the decision to reduce the sample from 16 women who are active on YouTube, to eight women who are active on both YouTube and Instagram (hereafter referred to as ‘social media users’) – thereby also excluding all inactive and the least active YouTubers from this study.

Despite the aim to be inclusive in terms of gender, the sample consists solely of women because no Dutch male YouTubers with a contested illness were identified. While the reasons for this are necessarily speculative, they are likely to be informed by the fact that contested illness is more prevalent among women – with an estimated 70% being female (Bülow, 2008b). In addition, Bülow (2008b) observes that men might find it harder to tell narratives about illnesses that are understood to be ‘female conditions’. Other reasons concern gender differences in social media use. In their study on YouTube vlogs, Molyneaux et al. (2008) found that women are prone to address personal experiences in their videos, whereas men post more videos about entertainment. On Instagram, women are more represented and active than men are (Sheldon and Bryant, 2016), and are more inclined to use the medium for the posting of selfies, to present themselves as attractive, and as belonging to a particular social group (Dhir et al., 2016). They also post more “emotional and positive hashtag descriptions” (Ye et al., 2018). These studies indicate that women are more skilled at telling personal stories, including stories about contested illness, and more inclined to use YouTube and Instagram to share these stories and to engage in communities of peers.

The eight women in this study were between 20 and 45 years old, and most were in their twenties. By the end of the study, on March 1st 2019, they had posted a total of 517 YouTube videos, and their subscription number ranged from 94 to over 12,000 with an average of 1,938 subscribers. On Instagram, their content consisted of a total of 13,077 posts, and their amount of followers ranged from 454 to over 14,000 with an average of 1635 followers. In addition to posts, Instagram also enables the display of ‘Stories’ that are made up of short fragments – consisting of (manipulated and/or visually enhanced) video, images or snippets – which can be seen by followers for a period of 24 hours. Due to the fleeting existence of Stories it is impossible to determine the exact amounts of Stories that were posted by the sample. Yet the fact that seven out of eight women posted stories with great regularity – sometimes as many as 20 fragments a day – indicates that the amount of Stories far exceeds the amount of Instagram posts. These numbers show that all the social media users post regular updates on their profiles.

Of the sample, five social media users accepted my interview request. Two social media users, both with a relatively large number of subscribers and/or followers, did not respond to email invitations and/or private messages on Facebook and Instagram. One social media user did initially agree to be interviewed but later cancelled due to her ill health and prolonged bed boundedness. While 30 vlogs were transcribed and coded, most of the data was analyzed online. This way attention could be given to the interactive and dynamic features of social media, for example, the sequence in which various content is posted, the accumulation of ‘likes’ and comments, changes in the descriptions of a YouTube video or Instagram post, etc. Despite the fact that the social media content of this study is openly accessible, the use of semi-structured interviews informs my decisions to use pseudonyms, and to exclude personal characteristics (e.g. age, ethnicity, illness) or screen shots of specific posts. All the quotes below are my translations from Dutch to English.

## Sharing experiences of contested illness on YouTube and Instagram

### Balanced positivity

The findings indicate that social media users have two core motivations for sharing their experiences of contested illness on YouTube and Instagram. First, they want to tell their story as a means of giving testimony. All the social media users share experiences of being disbelieved and poorly understood, and use YouTube and Instagram to gain wider recognition for their condition. Second, and related, social media users seek to help, support and inspire fellow-sufferers by documenting their illness trajectory. This is done by informing the viewer of the (dis)advantages of past and current treatments, medication and lifestyle adjustments, and by offering the viewer a diverse set of medical, psychological and spiritual insights, as well as by providing practical tips and tricks for counteracting illness-specific symptoms.

The dual motivation of testifying and wanting to inspire others is indicative of the imagined audience of the social media users. The content expresses a desire to raise awareness and to alter societal perceptions of contested illness by visualizing their suffering on YouTube and Instagram. Social media users are aware that their content is public, and many put in effort to disseminate their online videos and posts as widely as possible. However, the social media users readily acknowledge that the vast majority of their audience consists of fellow sufferers. Indeed, as Daisy explained in an interview: “it’s mainly fellow sufferers who follow me, and who recognize the posted content and get a lot of support from that”. While responses of “haters” are feared and anticipated, and do indeed occasionally occur, the vast majority of the social interactions are between the social media users and subscribers/followers who self-identify with the posted content.

In their online content, social media users put in effort not to be perceived as too negative. To mitigate (potential) accusations of coming across as gloomy and depressed, they take care to narrate their illness in a positive way. This is illustrated in the following interview excerpt, in which Nicole talks about her considerations in shooting her first YouTube video:

“Well, making that first video, I spent a lot of time on that. [. . .] And then I showed it to my partner, and he said: well, you sound a bit, a bit angry, and also very negative. And that is not what I wanted at all! [. . .] Because I do not want to come across as someone who is somber and negative. Because that’s not who I am [laughs]. *Sometimes*, of course.”

Interviewer: You could also say: that’s just who I am sometimes, so I put it online anyway. But that’s not what you thought.

No, because I don’t think it’s good to. . . when I put it online, that it’s all gloomy and negative. That might be interesting to people who have [my illness] as well: they can then watch it and become nice and depressed [laughing] - but that is not my intention!

Even though Nicole acknowledges that it can be challenging not to sound angry and negative in her YouTube videos, and that she is sometimes gloomy about her condition, she takes care to ensure that her audience perceives her content as positive.

However, being too positive is considered equally undesirable. The social media users readily state that YouTube and Instagram are filled with superficial influencers who post overly positive vlogs. In their narrations, they often contrast their own “authentic” content with the inauthentic content of these influencers. As Jane expressed: “the big vloggers on YouTube only show fun and nice things”. Conversely, her own aim is “to show that life can (unfortunately) look very different, and that that’s tough, but ok”. Along similar lines, Phoebe expressed her dislike for the seemingly effortless, yet highly manipulated content posted by some American celebrities: “then it’s no longer real and no longer of the person [themselves].” Lastly, Daisy recalls in an interview how she initially followed some native English-speaking vloggers (mainly from the United States), but soon stopped doing so because she considered them too extreme:

In the beginning, I followed [native] English stories and those were extremely negative, or extremely ‘wow, now it’s over!’ Not very realistic. [. . .] You don’t have to say ‘I am in this space and I learned something fabulous.’ No, [sometimes] it’s just what it is.

This shows how – in their aim to be more authentic – the social media users of this study seek to “disrupt the rule of positivity” (see Berryman and Kavka, 2018: 87) by illustrating how their lives revolve around managing the daily ups and downs inherent to contested illness.

The search for the right balance between being too positive and too negative is a central theme in the analyzed content. The social media users strive for what I describe as *balanced positivity*. This term captures how the social media users narrate contested illness in a predominantly positive way, as well as through the (occasional) display of hardship. By illustrating their investment in self-care practices, and by illustrating how symptoms persevere despite these practices, they seek to establish moral legitimacy. Three aspects are identified that are particularly relevant to the performance of balanced positivity, namely (1) appearances, (2) mind-set, and (3) presence. In the following three subsections, these aspects will be discussed in greater depth.

#### Practicing appearances: Looking nice, being ill

In their YouTube videos, the social media users take care to present themselves in a composed and feminine way. Particularly in introductions and in “talk vlogs”, in which social media users discuss a certain topic, they are usually cheerful and, often, wearing make-up. On some occasions, however, they also include footage on which they are not wearing make-up, their hair is unkempt and messy, and they are lying in bed or on the couch. Because including such footage is not conventional on YouTube, they take care to apologize for their looks and/or to explain the reasons for not having taken proper care of themselves – often by referring to persistent pain or a lack of energy.

Such footage also appears in the form of flashbacks. These are set apart from the remainder of the vlog through specific editing choices, such as a black-and-white color scheme and dramatic music. This way, the YouTuber creates a distinction between herself as a good-looking first-person narrator, and a third-person protagonist who is struggling and on whose life the narrator is commenting. On YouTube, then, moments of not looking nice are thus legitimized (and sometimes even apologized for) or are stylistically set apart from the remainder of the vlog through their incorporation as flashbacks.

Some of the social media users also post make-up tutorials, in which viewers are explained the best strategies for camouflaging apparent manifestations of chronic illness – such as a pale or patchy skin, bags under eyes or hair loss. Through these tutorials, these YouTubers give their viewers a look behind the scenes by addressing the continual effort involved in keeping up ‘normal’ appearances in their everyday lives. In addition, putting on make-up can be interpreted as a way of practicing self-care, and is illustrative of the social media users’ aim to show others how to manage chronic illness.

On Instagram, the tension between looking nice and being ill figures more prominently. Instagram prioritizes visual material; only when followers click on an image they see the ‘caption’ that describes or contextualizes a given image. As has become common practice among (semi-) professional Instagrammers (Marwick, 2015), posts by the social media users typically consist of selfies, portraits or impression shots, which are aesthetically enhanced through strategic cropping decisions and the use of filters (often ones that brighten or dramatize the photo, or transform it into a black-and-white image) (Manovich, 2017). While these posts conform to the “attention economy” of Instagram in the sense that they are visually appealing (Marwick, 2015: 138), chronically ill social media users need to put in effort to legitimate such stylized photos, so as to make them congruent with their illness narration. To do so (lengthy) captions are used to explain, contextualize or contrast the aesthetic quality of the post. Take a caption by Elisabeth, for example:

“I have worked very hard and still do, every day. But it gets rewarded. [. . .] I am proud to be here, and that, despite all the shit that came my way, I have become a stronger person who can tell her story to inspire you.”

This caption accompanies a portrait of Elisabeth standing against a white background in soft, romantic light, wearing make-up and smiling serenely. In text, she explains how her current happiness is the “award” for her work ethos. By adding this description to a stylized portrait, the implicit message of such posts is that good looks are not a given, but rather the result of individual motivation and self-discipline.

Most social media users also use Instagram Stories to broadcast ‘snippets’ or short video fragments. While these snippets have a maximum length of 15 seconds, most are shorter and last only 2–5 seconds. Content can be either text, stickers and/or emoticons, images, photos, short video fragments, or any combination of these. In terms of visual material, much of the content consists of photos and fragments that are produced by the social media user during daily activities, such as lying in bed or going for a walk. In addition to being a way to instantly report on illness-related experiences, Stories are used to share thoughts, doubts, hardship, and meaningful ideas and quotes with others who are chronically ill. As such, Instagram Stories gives the social media users an important means to balance the stylized and aesthetic quality of their Instagram posts:

“I am very active on Instagram. I like – because I share many tough things in my vlogs – to post nice photos that ‘feel’ a lot less sick. Of course there are people who don’t know you who respond: well you can’t be that sick, look how great you look. . . They don’t realize that it took a thick layer of make-up and several filters, and that after a photoshoot or trip I have seizures and need days to recover/of lying in a dark room. [. . .] But I also use Insta Stories to share my healing process, which goes with enormous ups and downs. So [. . .] a mix of positive and negative stories.” – Quote from interview with Jane

This different use of posts and Stories might be explained in reference to their temporality: while posts are meant to be permanent, Stories are fleeting in the sense that they are disappear after 24 hours (unless they are archived by the Instagrammer).

These examples illustrate how performing balanced positivity encompasses the need to navigate appearances. By limiting composed and stylized self-representations to video introductions and Instagram Posts, and by incorporating moments of not looking well in flashbacks and Instagram Stories, social media users make strategic use of the technological affordances to narrate their illness experiences. Through these particular engagements with the (temporal) features of YouTube and Instagram, they illustrate how contested illness is an important part of their self-representations, whilst also making clear that their identity cannot be reduced to their respective conditions. These examples also show how navigating appearances is a risky practice: while moments of ‘not looking good’ are illustrative of contested illness, they can also indicate a momentary lack of self-care. Conversely, stylized self-images can also be problematic because they can spark accusations that one is not truly sick at all. By showing their audiences the care they put into grooming and cultivating a feminine appearance, and also by showing how they are unsuccessful in doing so all the time, social media users seek to prove that they are truly ill. It allows them to illustrate how self-care practices are necessary, but ultimately insufficient in ensuring good-health.

#### Practicing mindset: Taking care, feeling bad

Another aspect of ‘balanced positivity’ is the narration of a right mindset. On both YouTube and Instagram, the social media users are occupied with cultivating, illustrating, and instructing others on the importance of properly dealing with chronic illness by adopting a right mindset. This mindset is conceptualized as something that can, and should, be cultivated. Indeed, content is filled with examples where the social media user explains to her audience what having a right mindset entails, as well as the kind of practices through which to express it. Examples of such practices include the mindful directing of attention to positive things, practicing gratitude by writing down what goes well in the morning, carefully planning one’s daily activities, being creative and enterprising, not doing too much, listening to body signals, staying in shape, making an effort by dressing stylishly and wearing make-up, eating healthy and taking specific supplements.

In addition, the social media users also emphasize that it is important to acknowledge feeling bad every now and then, and to be open about darker moments. On YouTube, such moments figure most prominently in ‘overview vlogs’, documenting a day or week of a social media user’s life. In such vlogs, the viewer is made aware of how much the life of the social media user evolves around dosing energy, limiting activities, and not doing too much. And whenever she overexerts herself, she shows her viewers how she is ‘punished’ for not listening to her body, and how this results in having a ‘bad day’ on which she is forced to recover by not doing anything at all. Occasionally such vlogs include overtly negative affect, for example when the social media user films herself when she is crying due to pain, frustration, or other setbacks. These expressions are affirmatively responded to by the viewers in comments, as well as through direct messages on other social media – most notably Instagram and Facebook – as explained by the social media users in interviews.

In Instagram posts, hardship is occasionally shown on stylized self-images, for example on selfies where mascara stains and a saddened expression indicate that the social media user is crying. However, hardship figures most prominently in Stories. This is illustrated by the following Instagram Story by Grace. On a pastel pink background, she writes in type machine typography:

“It might seem as though I’m doing all right. Lots of posts, lots of interactions with followers, and lots of positivity. But ‘behind the scenes’ it isn’t going as well as things might appear. What you see on insta is a small percentage of who I really am. It’s all real, very real. In the essence of my being I am cheerful, spontaneous, I love positivity and I am crazy about making online content. But there are many, many things that go past you. Not all is nice and fun. Every day is a battle. A battle against the Lyme disease that I have”

As is the case for half of the social media users, Grace’s illness-related content forms only a small portion of her overall content (which is mainly about lifestyle, fashion, and beauty). She is the only one who solely addresses it in Stories using text and emoticons on stock photo images. These Stories partially balance her ‘positivity’ by giving her followers a descriptive look “behind the scenes” into her everyday battle against Lyme disease. Similar to the previous section, the technological affordances of Instagram thus enable social media users to present themselves as coping well, whilst they also enable them to present moments of not coping as momentary disruptions that undermine their true self-identity.

Despite the fact that the social media users stress that their message is (and should be) one of positivity and perseverance, the measured display of negative affect is central to their online performances. Indeed, whenever content becomes too entrepreneurial, the audience responds to this with fewer views and likes, and sometimes even by an increase in dislikes. For example, Daisy, who used to make weekly overview vlogs, decided to diversify her content by posting travel vlogs, in which she would document her trips whilst informing the viewer of the adjustments made to cater to her chronic illness. While she greatly enjoyed editing and posting these vlogs, they were not “picked up, or responded to” by her followers, who preferred her weekly overview vlogs. Daisy, on the other hand, did not really enjoy making these weekly vlogs anymore:

“I noticed that, with those week vlogs, I would often have a really bad week and did not feel like editing, because that would confront me with that bad week, in which I was crying every day and was in pain all the time. But I know that my followers, they react to that especially, because they recognize it.”

Along similar lines, Jane stated that although she preferred looking back at vlogs about “fun trips”, her followers preferred seeing the “heavy sides”, such as the attacks she would have after doing something fun for a few hours: “that is where I get the most positive comments”.

In sum, most of the social media users’ online content focuses on (the cultivation of) self-care practices. They present themselves as women who work hard to manage and mitigate their respective conditions. Their content also illustrates how, despite these efforts, pain, fatigue, and setbacks continue to intrude upon their lives. Even though social media users do mention that they prefer presenting themselves in composed and optimistic manner, they also feel that showing setbacks and expressions of negative affect is equally constitutive of the stories they tell, and is particularly significant to their online communities. Through their online content, they illustrate how their positive mindset is the accomplishment of the continual effort they put into overcoming their bodily suffering.

#### Practicing presence: Seeking engagement, being absent

The last aspect of practicing ‘balanced positivity’ concerns the social media users’ online presence. In this study, all the social media users seek to actively engage in their online communities. Several have a background in journalism or marketing, and most practice their online activities in a semi-professional manner. They employ “self-branding strategies” (Marwick, 2013) such as posting regular status updates, using recognizable banners and formats, encouraging viewers to subscribe to their channel (on YouTube), and by promoting other types of content through the insertion of links in their videos and captions. Specific to Instagram is the use of hashtags, for example: #lymedontkillmyvibe, #fibrofighter, and #chronicillness. By adding these hashtags, posts show up in the search results of people looking for these specific topics. Lastly, the social media users take care to respond to most of the comments and messages – at the very minimum by replying affirmatively with a heart-emoticon. These practices illustrate the social media users’ effort to disseminate their content as widely as possible, and to engage in their online communities.

At the same time, the social media users frequently stress their inability to be as engaged as they would like to be. Rebecca’s story is illustrative in this regard: while she used to post frequently on YouTube, she was forced to recede from posting videos for four successive months due to prolonged ill health. During this time, her parents opened a crowdfunding page to raise money for a stem cell treatment in a private clinic in Germany, which they promoted on a newly made Facebook page for their daughter and on various popular media outlets. The cover picture of this Facebook page features photos of their daughter – probably selfies – that give the viewer the impression that she is severely ill. All the content on the Facebook page was posted by the parents. This transition from the regular posting of videos to (static) photos, and from the social media user’s own posts to her parents’, sparked alarm among her audience. Many of her subscribers and followers sent messages to Rebecca asking her how she was doing, and the requested amount of money for the stem cell treatment was soon exceeded. When she recommenced her online activities and posted a new YouTube video, her subscribers responded elatedly, and many expressed how glad they were of her recovery and return on social media. By welcoming her back on YouTube, they acknowledge the legitimacy of her periodic absence, thereby affirming her identity as chronically ill.

While the example is of a relatively long-term absence from social media, other examples are of short-term absences (e.g. days or weeks), the downscaling of posted content, the transitioning from color selfies to black-and-white selfies, or the switch from self-images to other images and text. Periodic breaks from social media are understood to require justification – especially when the social media user normally posts Instagram Stories daily. One way to do this is by addressing the period of absence in hindsight, for example by referencing prolonged illness or a lack of energy. Another way is by transitioning to a different type of images – such as stock photo images, pets, or landscapes – and adding captions in which the social media users explain their motivation for not posting a self-portrait. In the following interview quote, Phoebe explains how she posts pictures of her dog when she is too unwell to post a selfie:

“[My dog] is a bit of a lightning rod, because when I am feeling really, really too unwell to show myself I show [her]. That’s how I keep the story going.” – Interview quote by Phoebe

Lastly, the social media users sometimes post Instagram Stories that solely consist of written text and emoticons – most notably when symptoms seriously challenge the conducting of daily activities. Such intimate posts incite the audience to express their support and encouragement, evident from the fact that many of the social media users later post a thank-you note for all the ‘sweet’ and ‘reaffirming’ comments and direct messages.

These examples show how the social media users’ desire to be actively engaged in their online communities, as well as their openness about how their illness prevents them from being fully engaged, are central to the performance of balanced positivity. By using specific self-branding practices, social media users present themselves as members of an online community that focuses on norms of positivity and active coping. At the same time, their (periodic) absences function as proof of the fact that, despite their efforts and desires, their illness prevents them from being engaged more frequently. Yet being disengaged too frequently or for too long can also be counterproductive because of the social media platforms’ algorithmic prioritization of frequent-posting users. In addition, viewers themselves can lose their interest. As such, navigating ones online presence is, again, a precarious practice that social media users carefully navigate in their online narrations of contested illness.

## Discussion

This study shows how women narrate their experiences of contested illness on social media through *legitimacy narratives*. By documenting the effort involved in looking nice, being positive, and engaging in an online community, the narrator illustrates how – despite her continual efforts to cope with illness – chronic symptoms persevere. These findings resonate with Paxman’s (2019) study on fibromyalgia narratives, which, she argues, “refute the notion that hard work conquers all” by showing how life is subjected to a chronic, capricious and poorly understood condition (p. 14). Such stories voice how sufferers have struggled to overcome their condition, yet “they consistently conclude that total control of their disease is not possible” (p. 14). Paxman connects this finding to the American ideology of “hard work” and argues that fibromyalgia sufferers’ inability to comply with dominant discourses of productivity stands in the way of getting recognition.

In the present study, the understanding that the imperative of “hard work” in fibromyalgia narratives is America-specific is challenged by illustrating how Dutch narratives of contested illness are equally entangled with neoliberal ideals of healthism (Lupton, 2018). This study also draws attention to the fact that, on social media, the understanding that contested illness cannot be controlled is not a “conclusion” of the stories that women with contested illness tell. Rather it is a message that is invoked repeatedly and persistently throughout their online content. On a related note, I argue that the legitimacy narratives of social media users both affirm and challenge the contemporary imperative of healthism. They affirm it by illustrating how practices of self-care are adopted to mitigate and manage contested illness. As has become apparent, the online narrations of women with a contested illness are premised on the understanding that moral legitimacy is granted to those who illustrate (a desire for) active coping and productivity. It is likely that these narrations have a disempowering effect on viewers who are too sick to take care of themselves even minimally, because the implicit message in these narrations is that sufferers of contested illness are morally obliged to engage in practices of self-care. Conversely, the narrations also challenge the imperative to health by showing how contested illness continues to affect their lives despite their efforts. This presents a paradox: to challenge the claim that good health is the result of self-management and individual dedication, the social media users in this study are compelled to show others how they themselves individually engage in a range of self-care practices. As such, the imperative of health is simultaneously challenged and endorsed.

Lastly, what this study illustrates is that, even though social media content is publicly accessible and actively disseminated, most of the social media users’ interactions are with followers and subscribers who belong to the same community. Characterizing illness experience as an “increasingly public affair” (Conrad et al., 2016) does not capture the intricacies of ‘sharing’ illness online. Rather, these experiences can better be described as a “public-yet-situated affair”. This characterization recognizes that social media offer new and more public ways to share illness-related experiences with others, whilst also acknowledging that sharing illness on social media is characterized by the disclosure of illness-related experiences within the context of, and in interaction with, the members of situated online communities.

## Conclusion

This study shows how narrating contested illness on social media is a delicate performance that involves a continual balancing between being positive, but not too positive. Sufferers of contested illnesses such as CPS, CFS, and CLD need to put in a great deal of effort to be perceived as credibly ill. Without a clear biomedical diagnosis, they are easily subjected to moral accusations in which the sufferer is held (partially) accountable for her prolonged ill health. On social media, we see how practicing *balanced positivity* enables narrators with a contested illness to voice their experiences in a way that serves to affirm their moral legitimacy. This involves showing their personal investment in self-care practices – by looking good, being positive, and involved in a community – whilst also illustrating how chronic symptoms nevertheless persevere. Balancing this is done by showing both positive self-care practices as well as the negative effects of their chronic illness, and by making strategic use of the technical, editorial, and temporal features of YouTube and Instagram. This way, sharing stories of contested illness on social media functions as a way to establish moral legitimacy, and to articulate oneself as someone “whose story deserves to be told” (Japp and Japp, 2005: 112). Future research must gain a more thorough understanding of how viewers and followers engage with social media content about contested illness, as well as the extent to which they feel adequately represented by it.

## References

[bibr1-13634593211017187] Barker KK (2008) Electronic support groups, patient-consumers, and medicalization: The case of contested illness. Journal of Health and Social Behavior 49: 20–36. 18418983 10.1177/002214650804900103

[bibr2-13634593211017187] Berard AA Smith AP (2019) Post your journey: Instagram as a support community for people with fibromyalgia. Qualitative Health Research 29(2): 237–247. 30066603 10.1177/1049732318789102

[bibr3-13634593211017187] Berryman R Kavka M (2018) Crying on YouTube: Vlogs, self-exposure and the productivity of negative affect. Convergence: The International Journal of Research into New Media Technologies 24(1): 85–98.

[bibr4-13634593211017187] Bülow P (2008a) Tracing contours of contestation in narratives about chronic fatigue syndrome. In: Moss P Teghtsoonian K (eds) Contesting Illness: Process and Practices. Toronto, Canada: University of Toronto Press, pp.123–141.

[bibr5-13634593211017187] Bülow P (2008b) “You have to ask a little”: Troublesome storytelling about contested illness. In: Hydén L-C Brockmeier J (eds) Health, Illness and Culture: Broken Narratives. London: Routledge, pp.131–153.

[bibr6-13634593211017187] Conrad P Bandini J Vasquez A (2016) Illness and the internet: From private to public experience. Health 20(1): 22–32. 26525400 10.1177/1363459315611941

[bibr7-13634593211017187] Crawford R (1980) Healthism and the medicalization of everyday life. International Journal of Health Services 10(3): 365–388. 7419309 10.2190/3H2H-3XJN-3KAY-G9NY

[bibr8-13634593211017187] Dhir A Pallesen S Torsheim T , et al. (2016) Do age and gender differences exist in selfie-related behaviours. Computers in Human Behavior 63: 549–555.

[bibr9-13634593211017187] Dumit J (2006) Illnesses you have to fight to get: Facts as forces in uncertain, emergent illness. Social Science & Medicine 62(3): 577–590. 16085344 10.1016/j.socscimed.2005.06.018

[bibr10-13634593211017187] Frank A (1995) The Wounded Storyteller. Chicago, IL: University of Chicago Press.

[bibr11-13634593211017187] Japp PM Japp DK (2005) Desperately seeking legitimacy: Narratives of a biomedically invisible disease. In Harter LM Japp PM Beck CS (eds) Narratives, Health and Healing: Communication Theory, Research, and Practice. Mahwah, NJ: Erlbaum, pp.107–130.

[bibr12-13634593211017187] Keim-Malpass J Steevens RH Kennedy C (2014) Internet ethnography: A review of methodological considerations for studying online illness blogs. International Journal of Nursing Studies 51: 1686–1692. 24985757 10.1016/j.ijnurstu.2014.06.003

[bibr13-13634593211017187] Kristensen DB Lim M Askegaard S (2016) Healthism in Denmark: State, market, and the search for a “Moral Compass”. Health 20(5): 485–504. 27000136 10.1177/1363459316638541

[bibr14-13634593211017187] Lantos PM (2015) Chronic Lyme disease. Infectious Disease Clinics of North America 29(2): 325–340. 25999227 10.1016/j.idc.2015.02.006 PMC4477530

[bibr15-13634593211017187] Lupton D (1995) The Imperative of Health: Public Health and the Regulated Body. London, England: Sage.

[bibr16-13634593211017187] Lupton D (2018) Digital Health: Critical and Cross-Disciplinary Perspectives. London, England: Routledge.

[bibr17-13634593211017187] Malterud K (2000) Symptoms as a source of medical knowledge: Understanding medically unexplained disorder in women. Family Medicine 32(9): 603–611. 11039146

[bibr18-13634593211017187] Manovich L (2017) Instagram and contemporary image. [Book in online PDF]. Available at: http://manovich.net/content/04-projects/154-instagram-and-contemporary-image/instagram_book_manovich_2017.pdf (accessed 17 May 2021).

[bibr19-13634593211017187] Marques A (2008) Chronic Lyme disease: An appraisal. Infectious Disease Clinics of North America 22(2): 341–360. 18452806 10.1016/j.idc.2007.12.011 PMC2430045

[bibr20-13634593211017187] Marwick AE (2015) Instafame: Luxury selfies in the Attention Economy. Public Culture 27(1): 137–160.

[bibr21-13634593211017187] Marwick AE (2013) Status Update: Celebrity, Publicity, and Branding in the Social Media Age. New Haven, CT: Yale University Press.

[bibr22-13634593211017187] Marwick AE Boyd D (2010) I tweet honestly, I tweet passionately: Twitter users, context collapse, and the imagined audience. New Media & Society 13(1): 114–133.

[bibr23-13634593211017187] McMahon L Murray C Sanderson J , et al. (2012) “Governed by the pain”: Narratives of fibromyalgia. Disability and Rehabilitation 34(16): 1358–1366. 22263672 10.3109/09638288.2011.645114

[bibr24-13634593211017187] Molyneaux H O’Donnell S Gibson K , et al. (2008) Exploring the gender divide on YouTube: An analysis of the creation and reception of vlogs. American Communication Journal 10(1): 1–14.

[bibr25-13634593211017187] Nettleton S (2006) ‘I just want permission to be ill’: Towards a sociology of medically unexplained symptoms. Social Science & Medicine 62: 1167–1178. 16135395 10.1016/j.socscimed.2005.07.030

[bibr26-13634593211017187] Østebye SV Kvamme MF Wang CE , et al. (2018) ‘Not a film about my slackness’: Making sense of medically unexplained illness in youth using collaborative methods. Health 24: 38–58. 29978724 10.1177/1363459318785696

[bibr27-13634593211017187] Paxman C (2019) “Everyone thinks I am just lazy”: Legitimacy narratives of Americans suffering from fibromyalgia. Health 25: 121–137. 31216871 10.1177/1363459319857457

[bibr28-13634593211017187] Phillips T Rees T (2017) (In)visibility online: The benefits of online patient forums for people with a hidden illness: The case of multiple chemical sensitivity (MCS). Medical Anthropology Quarterly 32: 214–132. 10.1111/maq.12397 28726292

[bibr29-13634593211017187] Poletti A Rak J (2014) Introduction: Digital technologies. In Poletti A Rak J (eds) Identity Technologies: Constructing the Self Online. Madison, WI: University of Wisconsin Press, pp.3–22.

[bibr30-13634593211017187] Sannon S Murnane EL Bazarova NN , et al. (2019) “I was really, really nervous about posting it”: Communicating about invisible chronic illnesses across social media platforms. In: CHI conference of human factors in computing systems proceedings (CHI 2019), Glasgow, Scotland, 4–9 May 2019. New York: ACM.

[bibr31-13634593211017187] Sheldon P Bryant K (2016) Instagram: Motives for its use and relationship to narcissism and contextual age. Computers in Human Behavior 58: 89–97.

[bibr32-13634593211017187] Sim J Madden S (2008) Illness experience in fibromyalgia syndrome: A metasynthesis of qualitative studies. Social Science & Medicine 67: 57–67. 18423826 10.1016/j.socscimed.2008.03.003

[bibr33-13634593211017187] Sowińska A (2018) ‘I didn’t want to be Psycho no. 1’: Identity struggles in narratives of patients presenting medically unexplained symptoms. Discourse Studies 20(4): 506–522.

[bibr34-13634593211017187] Swoboda DA (2006) The social construction of contested illness legitimacy: A grounded theory analysis. Qualitative Research in Psychology 3: 233–251.

[bibr35-13634593211017187] Van Dijck J (2009) Users like you? Theorizing agency in user-generated content. Media, Culture and Society 31(1): 41–58.

[bibr36-13634593211017187] Wasson S (2018) Before narrative: Episodic reading and representations of chronic pain. Medical Humanities 44: 106–112. 29305389 10.1136/medhum-2017-011223 PMC6031271

[bibr37-13634593211017187] Werner A Isaksen LW Malterud K (2004) ‘I am not the kind of woman who complains of everything’: Illness stories on self and shame in women with chronic pain. Social Science & Medicine 59: 1035–1045. 15186903 10.1016/j.socscimed.2003.12.001

[bibr38-13634593211017187] Werner A Malterud K (2003) It is hard work behaving as a credible patient: Encounters between women with chronic pain and their doctors. Social Science & Medicine 57: 1409–1419. 12927471 10.1016/s0277-9536(02)00520-8

[bibr39-13634593211017187] Willemsen M (2018) Leven met lyme–of is het een andere ziekte? [Newspaper article NRC]. Available at: https://www.nrc.nl/nieuws/2018/11/06/leven-met-lyme-of-is-het-een-andere-ziekte-a2754180 (accessed 17 May 2021).

[bibr40-13634593211017187] Ye Z Hashim NH Baghirov F , et al. (2018) Gender differences in Instagram hashtag use. Journal of Hospitality Marketing and Management 27(4): 386–404.

